# Influence of Physical Activity and Socio-Economic Status on Depression and Anxiety Symptoms in Patients after Stroke

**DOI:** 10.3390/ijerph18158058

**Published:** 2021-07-29

**Authors:** Małgorzata Paprocka-Borowicz, Mona Wiatr, Maria Ciałowicz, Wojciech Borowicz, Agnieszka Kaczmarek, Adilson Marques, Eugenia Murawska-Ciałowicz

**Affiliations:** 1 Department of Physiotherapy, Physiotherapy Faculty, Medical University in Wroclaw, 50-355 Wroclaw, Poland; malgorzata.paprocka-borowicz@umed.wroc.pl (M.P.-B.); mona.wiatr@gmail.com (M.W.); 2 Physiotherapy Faculty, University School of Physical Education, 51-612 Wroclaw, Poland; marysia.cialowicz@gmail.com; 3 Neurological Diseases Department, Medical University in Wroclaw, 51-618 Wroclaw, Poland; wojciech.borowicz@student.umed.wroc.pl; 4 Physiology and Biochemistry Department, University School of Physical Education, 51-612 Wroclaw, Poland; agnieszka.kaczmarek@awf.wroc.pl; 5 CIPER, Faculty of Human Kinetics, University of Lisbon, 1499-002 Cruz Quebrada, Portugal; amarques@fmh.ulisboa.pt

**Keywords:** stroke, post-stroke depression, exercise, physical activity, kinesiotherapy, socio-economic status

## Abstract

Stroke is a high-risk factor for depression. Neurological rehabilitation is greatly difficult and often does not include treatment of depression. The post-stroke depression plays an important role in the progress of treatment, health, and the life of the patient. The appropriate treatment of depression could improve the quality of life of the patient and their family. The study aimed to evaluate the impact of physical activity and socio-economic status of the patient on the effectiveness of recovery from depression and the severity of the symptoms of depression. The study was conducted with 40 patients after stroke aged 42–82 years, and included 10 women and 30 men who were hospitalized for two weeks. The severity of depression/anxiety (D/A) symptoms were evaluated two times; at admission and after two weeks of physical therapy. The hospital anxiety and depression scale (HADS) questionnaire was used for this purpose. Socio-economic status was evaluated by several simple questions. It was revealed that physical therapy has a positive influence on mental state. The severity of D/A symptoms after stroke is related to the financial status of the patients (χ^2^ = 11.198, *p* = 0.024). The state of health (χ^2^ = 20.57, *p* = 0.022) and physical fitness (χ^2^ = 12.95, *p* = 0.044) changed the severity of symptoms of anxiety and depressive disorders. The kinesiotherapy in the group of patients with post-stroke depression had positive effects; however, economic and health conditions may influence the prognosis of the disease.

## 1. Introduction

Stroke is the third cause of death in the world and is one of the most commonly occurred diseases of the nervous system [1,2]. It is also a somatic disease burdened with a high risk of depression [3]. In patients after stroke, most attention is focused on restoring mobility in the therapeutic process. The therapy strives at all costs to achieve the best possible physical ability and independence in the patient’s daily responsibilities. Therefore, the significant complication of a psychological nature, including depression is moved to the background.

Increasingly, the opinion is held that post-stroke depression is crucial in the forecast of a patient’s life and health. It significantly hinders and delays the progress of rehabilitation, prevents the patient from achieving the appropriate therapeutic goals, and negatively impacts the process of social adaptation. Ultimately it decreases the quality of not only the patient’s life but also their caregivers [3,4,5]. Despite the growing interest in the problem of post-stroke depression, the diagnosis of this disease remains difficult. Many patients are not diagnosed, in some cases a diagnosis is too late, or improperly treated. Very often performing an appropriate diagnosis is difficult due to the presence of atypical symptoms and the necessity of performing a diagnosis, which differentiates depression from many other mood disorders (dementia, apathy) [4].

Among the neuropsychiatric disorders that occur in patients after a stroke incident, post-stroke depression (PSD) requires special attention. It is symptomatic depression, occurring as a result of somatic diseases of the organism. Along with other types of depression, it is diagnosed more often in women [3,6,7]. The prevalence of post-stroke depression is estimated at 17–65% of patients and depends on the clinical course of the disease. Methodological differences in the conducted studies and a large number of research tools lead to diverse results in the assessment of the incidence of post-stroke depression [5]. The criteria relating specifically to post-stroke depression have not been specified, and numerous questionnaires and scales used for screening tests do not take into account communication disorders in a patient (aphasia) or cognitive disorders [3,8,9]. The duration of post-stroke depression is estimated at 7–9 months, but it may occasionally extend up to 7 years [6,7]. Morris et al. [10] and others [11] have noticed that the occurrence of post-stroke depression increases the risk of death within the following 10 years by more than 3.4 times.

Among the factors determining the degree of depression, three of them can be considered as proven and undeniable. These are the severity of the stroke, the degree of physical disability resulting from the stroke, and the coexistence of cognitive disorders [5,8]. Indicators of social isolation, poor material, and social situation, and positive medical history in terms of family burden of affective diseases can be treated as relatively well-substantiated. Since it is believed that lonesome patients living in poor housing conditions show a higher level of depression and more often stop receiving medical services and rehabilitation treatments [12,13].

Post-stroke depression causes great diagnostic and therapeutic difficulties [10,14]. It is also the cause of significant deterioration in the daily activity of a patient, essentially decreasing the quality of his life. The negative influence of post-stroke depression on the course and results of the rehabilitation in patients after a stroke incident was also proven. The more severe the form of depression the worse the results of rehabilitation. In patients diagnosed with post-stroke depression, the process of physical recovery is inhibited and slow [3,5]. Patients with remission of symptoms of depression achieve improved results in scales that assess their coping with daily activities.

Despite the growing interest in the subject of post-stroke depression, this disease often remains undiagnosed and, as a result, not treated [15,16]. Due to the high probability of depression in patients after stroke, these patients’ mental state monitoring ought to be a mandatory part of the treatment. The interest in the topic of the impact of physical activity on the reduction/elimination of depression symptoms constantly increases; however, studies do not clearly define its positive influence. This is mainly due to numerous methodological limitations and diagnostic difficulties [17]. According to the researchers, it is not only physical activity itself that helps support the treatment of depression, but mainly its specific type. According to Palusk and Schwenk [18], the best form of physical activity is aerobic and stretching exercises. In this type of exercise, regularity and the correct technique execution count, not the load value. Although kinesiotherapy may be considered as an effective method of depression treatment, due to the complexity of the problem of depression, the best results are achieved by using psychotherapy and pharmacological treatment simultaneously [19,20].

Kinesiotherapy performed in patients after stroke not only alleviates the symptoms of depression but also improves the general physical prowess and patients’ daily life functioning. All the principles of comprehensive therapy and care for a patient after a stroke have been collected in the Helsingborg Declaration (1995) [21]. According to this document, rehabilitation ought to be undertaken in patients as early as 1–2 days after the onset of symptoms and with the participation of a cooperating rehabilitation team, it should last as long as the patient’s health condition requires.

Regular physical exercise supports improving life parameters and increases the efficiency of the body’s physical performance, improves well-being, and reduces pain. Improving the condition of the patients and teaching them how to cope with everyday duties allows them to achieve independence and independence in daily functioning [22]. During physical activity, happiness hormones or endorphins are released. They are peptide hormones classified as endogenous opioids, which relieve pain and enhance well-being. In depressive disorders, dysfunction of the serotoninergic system and a reduction in the amount of secreted neuromediators are found [23]. On the other hand, during physical activity, the concentration of serotonin in the brain increases, which provides a feeling of general satisfaction. Additionally, with intense physical exertion, body temperature rises by 3 °C, which reduces muscle tension, and thus mental tensions. Regular exercise of moderate intensity also reduces the reactivity of the hypothalamic-pituitary-adrenal system [24].

Regularly dosed exercise is an effective method of supporting the treatment of depression in patients who have had a stroke [17,18]. The financial and health situation also affects the risk of developing depression and the degree of its manifestation after a stroke.

Based on our own clinical experience and few reports, it can be concluded that the effectiveness of post-stroke physiotherapy depends on the patient’s mood and the severity of anxiety and depression symptoms. We hypothesized that, in addition to post-stroke functional disability, socio-economic factors such as living status, education, marital status, pay, and employment status can also influence and alter post-stroke mood and symptoms of anxiety/depression.

In order to investigate the impact of physical activity in the form of kinesiotherapy and the impact of socio-economic factors on the severity of anxiety/depression symptoms, two research goals were set: (i) to investigate the impact of 2-week kinesiotherapy on the course of depression in post-stroke patients (ii) and to investigate the impact of the financial situation and health care on the risk of developing the disease and the degree of depression in post-stroke patients.

## 2. Materials and Methods

### 2.1. Participants

The study included a group of 40 post-stroke patients hospitalized in the department of neurological rehabilitation. The criteria for inclusion in the study were: the patient’s consent in writing to participate in the study, a history of stroke-only patients after the first stroke, undergoing post-stroke rehabilitation, hospitalized for the first time, patients with no previous motor dysfunction, participation in exercise tests, no psychiatric disorders before the stroke, with diabetes, participation in the treatment of at least 2-weeks of hospital rehabilitation, and the patient’s independent and conscious response to the questions asked. The characteristics of health, family and economic status are presented in Table 1. The following patients were excluded from the study: those taking antidepressants, patients with aphasia or impaired consciousness, and patients who were not diagnosed with depression based on the hospital anxiety and depression scale (HADS) questionnaire. Patients were hospitalized for various periods. For the study, only the initial 2 weeks of rehabilitation were analyzed. During this time, each patient went through 12 treatment days, from Monday to Saturday, with a break on Sunday.

The research had a pilot nature. The patients were selected randomly from among the patients admitted to the ward. Socio-economic and clinical data as well as HADS scores were selected for the analysis. The study covered 10 (25%) women and 30 (75%) men. The age range in the study group was 42–82 years (mean 64 ± 14.98 years); height 167.63 ± 7.99 cm; body mass 73.64 ± 11.55 kg; and body mass index (BMI) 26.06 ± 2.53 kg/m^2^. Women hospitalized for stroke were 60–82 years old (mean 73 ± 7.56 years); height 169 ± 4.83 cm; body mass 75.20 ± 9.80 kg; body mass index (BMI) 26.44 ± 2.96 kg/m^2^ and men 42–73 years (mean 61 ± 7.43 years) height 167.33 ± 8.85 cm; body mass 73.13 ± 12.16 kg; and body mass index (BMI) 25.94 ± 2.41 kg/m^2^.

Only stroke patients participated in the studies. There was no control group as our highest goal was patient well-being. The process of post-stroke rehabilitation is a specific form of therapy, not used in other diseases and every patient has the right to it, therefore the control group was abandoned.

### 2.2. Measures

On admission to the hospital, each patient was asked to complete the Hospital Anxiety and Depression Scale (HADS) questionnaire and the poll and re-completed them after 2 weeks. The HADS scale is a commonly used tool for the initial assessment of depression and anxiety symptoms. It belongs to the group of self-assessment scales. The HADS scale is used for screening tests to assess negative emotions in elderly people, including people after a stroke incident. The entire questionnaire consists of 14 questions. The questionnaire has two subscales to evaluate anxiety and depression. Each subscale consists of seven questions/statements. Answers are given on a 4-point Likert scale (0–3 points). The final score for each subscale is in the range of 0–21 points. The result of the examination is the sum of the points obtained by the patient, counted separately. The questions are divided into two parts, the first of which assesses anxiety disorders, the second assesses depressive disorders. Points earned in each part are counted separately [25]. In the presented research, the points obtained from the questionnaire were interpreted as follows: 0–7, norm; 8–10, mild anxiety/depressive disorders; 11–14, moderate anxiety/depressive disorder; 15–21, severe anxiety/depressive disorders (A/D). In our study, we used point 7 as a threshold to identify underlying disorders. In patients with a history of stroke, it is suggested that the scale points should be lowered and 7 points should be considered as the threshold for determining the occurring disorders [26,27]. In recent years, the effectiveness of the HADS scale was assessed and confirmed in a Norwegian study [11,28]. The HADS scale is used in many centers, and is generally available, validated and translated into many languages. It is a clear scale for assessing the severity of anxiety/depression symptoms. This underlines its usefulness and ease of use. The authors wanted to use a simple, available tool that would allow comparisons of outcomes between stroke centers. By using this scale, they can help therapists compare the results.

### 2.3. Socio-Economic Status

The present study also investigated the socio-economic situation of patients. A questionnaire consisting of 14 questions concerning the economic status and patient’s concerns was used.

Patients were asked about their living conditions, education status, salary (in relation to the national average), social environments they lived (private houses, flats in municipal blocks and what floor), marital status, if they were living alone or with family, salary and employment status, how they assessed their physical fitness, what were they afraid of, could they count on family support, what made it difficult for them to function, whether patients had emotional or depressive disorders before the stroke, or frequent mood swings, how they assessed their current health condition. The responses were taken once upon admission to the hospital, the questionnaire lasted about 5–10 min and was dependent on the patient’s state of health. All answers received are presented on Table 2.

### 2.4. Rehabilitation Training

Before starting the rehabilitation training (kinesis), each patient was tested with the Lovett test. The Lovett test is currently the most common method of subjective muscle strength assessment. It involves the palpation of individual muscles in certain body positions and movement. In every movement, there is one main muscle that is responsible for movement. Due to the appropriate position, it is isolated from other synergistic muscles. According to Lovett, the following levels of muscle strength are distinguished: 0, no active muscle contraction; 1, a trace of active muscle contraction; 2, a clear muscle contraction and the ability to move and relieve mobility in a section; 3, the ability to move independently active with overcoming the weight of a given section; 4, the ability to perform active movement with some resistance; 5, correct force, i.e., the ability to perform active movement with full resistance. This scale can be expressed as a percentage, then: 0 = 0%, 1 = 10%, 2 = 25%, 3 = 50%, 4 = 75%, 5 = 100%. For a more accurate assessment of muscle strength, the intermediate steps 2+, 3 are used when strength is rated above 1° and below the next step.

Depending on the results obtained on the Lovett scale, patients were subjected to local or general kinesiotherapy. Local kinesiotherapy does not involve the whole organism, it affects the area of dysfunction, usually it does not change the circulatory and respiratory parameters, it can be used in people for whom exercise is contraindicated. It is conducted individually, we include the following exercises: passive, active-passive, self-assisted, active with unloaded, slow active, active with resistance exercises and other forms of exercise and interaction. Additionally, the patients also received general and relaxation kinesiotherapy and breathing exercises.

The frequency of exercises was set at five times a week. Each rehabilitation unit lasted 45 min, and two training units were used daily, i.e., 90 min of individual kinesiotherapy under the supervision of a physiotherapist. The daytime therapy sessions were broken down as follows: 45 min of exercise, 30 min of rest, another 45 min of exercise. Throughout the entire session, HR was recorded using a POLAR m400 sportster device (Kempele, Finland). The intensity of exercise was determined individually depending on the patient’s abilities and limitations and did not exceed 50–60% of the maximum heart rate (HRmax) determined according to the formula HRmax = 220-age. The physical training lasted two weeks and the symptoms of anxiety and depression were assessed after this time.

To carry out this study we have received the written consent of each patient of the study and also a Bioethics Committee Agreement of the Medical University in Wroclaw, Poland (number KB-672/2014).

### 2.5. Statistical Analysis

The test results were analyzed statistically. The characteristics of the studied group of patients, as well as socio-economic and clinical data, are presented by providing descriptive statistics. The Wilcoxon’s test was used to analyze if physical therapy has an impact on a patents’ psychical condition. The association between qualitative variables was studied using the χ^2^. The χ^2^ test was used for analysis of relation between financial status and severity of D/A symptoms. The Statistica 13.0 (StatSoft Inc., Tulsa, OK, USA) program was used to compile the statistical data. The difference was considered statistically significant when *p* < 0.05.

## 3. Results

The clinical data of the respondents are presented in Table 1. The studies assessed the location of the stroke in patients with symptoms of depression and/or anxiety disorders. This was noticed because some sources report that damage to the left hemisphere of the brain may predispose to a more frequent occurrence of depressive disorders. According to the conducted analysis, the prevalence of left-hemispheric lesions was observed among patients with mood disorders. Among the studied patients there were 29 (72.5%) left-sided and 11 (27.5%) right-sided stroke patients.

In the studied group of patients, the period from the stroke incident to the time of the study was also checked to assess the incidence of early and late post-stroke depression. Early depression was considered to be symptoms occurring within 6 months of the onset of stroke, and late more than a year. As many as 32 patients (80%) had early depression on the initial screening tests using the HADS scale, while only eight (20%) had late post-stroke depression. The group of patients in the early stage of post-stroke depression was predominant. It may, however, result from the overall greater number of patients in the regenerative-compensatory period of stroke staying in the hospital ward during the conducted examinations.

In the survey, patients were asked about their financial situation. Ten of the respondents (25%) described their financial situation as good, 17 (42.5%) consider it satisfactory, and 13 (32.5%) as bad. The examined patients also had to determine their current health condition. Among the possible answers, half of the respondents (20 people) described it as average, six as good, nine as satisfactory, and five as bad. Nobody rated their health as very good. The family situation was assessed next. The vast majority of patients can count on support and help from family, as many as 33 (82.5%), while seven (17.5%) do not receive much help from their relatives. The survey also revealed numerous fears faced by stroke patients daily. Most (15 people) are afraid of losing physical fitness, and 13 people are afraid of the lack of independence in performing daily activities. Although some patients do not receive support from their family, the survey shows that they are rather not afraid of the lack of help from their surroundings. Only one person provided such an answer. The patients’ socio-economic status data are presented in Table 2.

In the measured group, 37.5% of patients were higher educated, almost the same group, 35% had secondary education. Only 27.5% had primary level of education. Most patients lived with their families (75%), only 25% of patients lived alone. About a third of the sample (35%) lived in their private houses, the rest of the patients lived in municipal blocks of flats (65%), and the majority of responders on the third floor or higher. A total of 28 (70%) patients lived with a life partner; however, 12 (30%) lived alone without a partner. Among people who lived alone, two persons lived in their private houses and eight persons in blocks of flats. One of them has a flat on the ground floor, the other one on the third floor and without a lift. The financial status of patients despite the positive attitudes presented was as follows. Twenty-one persons (52.5%) earned at the level of the national average, the salary of 14 persons was above the national average and of five persons (12.5%) below average.

### 3.1. Assessment before and after Kinesiotherapy

**Figure 1:**
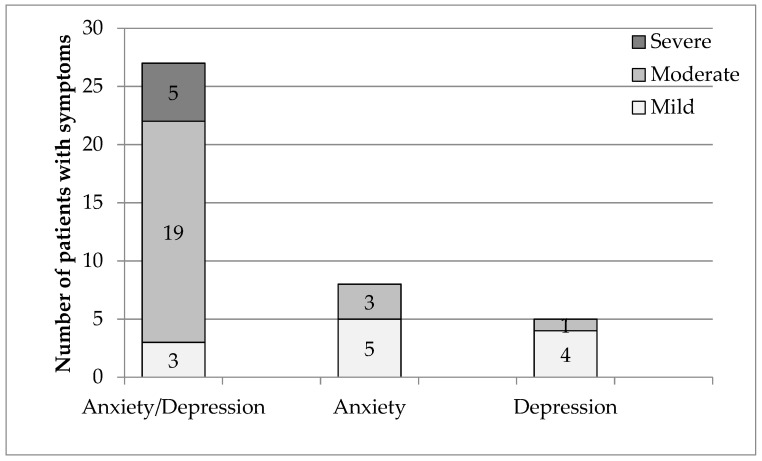
Type of disorders and their degree before therapy.

Before therapy the most numerous group were patients showing moderate symptoms of depression and/or anxiety disorders (A/D), and men predominated in this group. The next group in terms of numbers was mild symptomatic patients, only men with severe symptoms of depression (Figure 1).

The group was also characterized in terms of the type of diagnosed disorders. As many as 67.5% (27 patients) (Table 3) had both anxiety and depressive disorders (A-D), 20% had anxiety disorders (A) (8 patients), and 12.5% depressive disorders (D) (5 patients). The obtained results confirm that depressive and anxiety disorders most often coexist.

There were as many as 19 patients in group A/D with a moderate degree and three with a mild degree. The opposite was true for patients with anxiety or depressive disorders. In these groups, patients with a moderate degree of disturbances predominated (Figure 1, Table 3).

**Figure 2:**
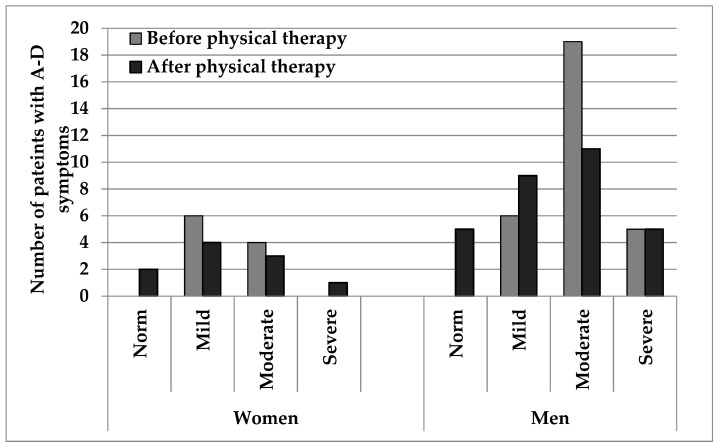
Degree of anxiety/depressive disorder before and after physical activity therapy by sex.

After 2 weeks of rehabilitation, some changes in the mental health of the studied patients were noticed. In seven patients with low degree of depression and/or anxiety disorders registered before the therapy, no symptoms of any disorders were found after rehabilitation (Figure 2). Patients with symptoms of moderate depression and/or anxiety disorders remained the most numerous group, but their number decreased from 23 to 15 people. Among the people showing symptoms of disorders, there was a decrease in the number of patients in the group of women by two and an increase in the number of men by three people. Previously, they were all men, and after the therapy five of the same men and additionally one woman. Thus, the intensification of anxiety and depression symptoms was observed in two people. In the greatest number of people, i.e., 22 (55%), no changes in symptoms of anxiety and/or depression were observed, in 16 (40%) an improvement in mood was observed, and only two (5%) had worsened symptoms (Figure 2).

### 3.2. Compare the Results of the First and Second Tests

Based on the data obtained, it can be seen that the mean levels of anxiety and depression fell by 1 point (Table 4). A decrease in the maximum values was observed after rehabilitation, as no one in the group scored 17 points on the HADS scale. Due to the small number of subjects, it was decided not to divide the whole group in terms of sex to assess the impact of physiotherapeutic rehabilitation and statistical analysis.

First, it was checked whether the improvement in anxiety recorded in the HADS scale was statistically significant. The distribution of variables obtained after assessing patients using the HADS scale did not show a feature of normality, therefore the significance level was tested using the Wilcoxon variable pair test. The statistical analysis shows that physiotherapeutic rehabilitation improved the condition of patients with anxiety disorders (*p* < 0.001). An improvement in depressive disorders was noted. After the initial analysis of the distribution and the application of the Wilcoxon test, it was found that rehabilitation also improved the condition of patients with depressive disorders (*p* = 0.007).

**Figure 3:**
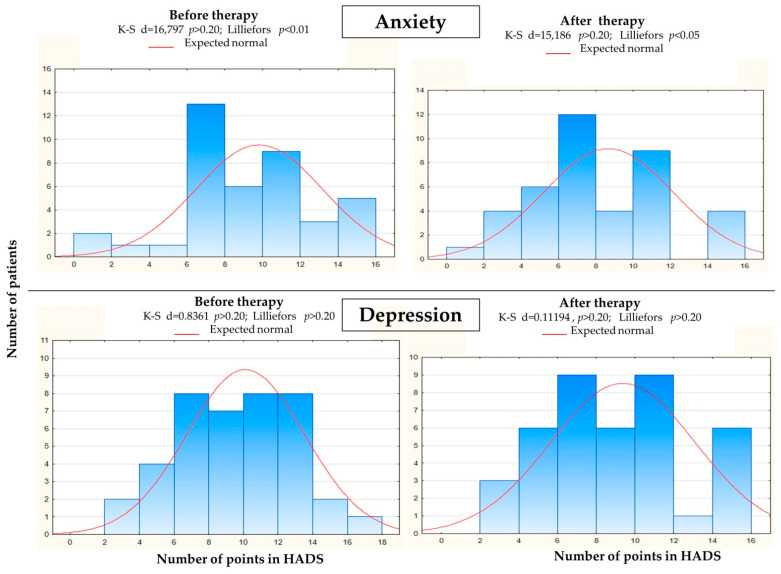
Histogram of HADS points in anxiety and depression disorders in measured group before and after therapy.

The next step was to check the changes in anxiety and depressive disorders, taking into account the severity of the disorders, using a multi-way table. The results obtained according to the HADS scale improved in 16 people (40%), in 22 people (55%) there were no significant changes, while the mental state of 2 people (5%) worsened (Figure 3). After 2 weeks of physiotherapeutic rehabilitation, no change was noted in 14 patients. These patients showed moderate symptoms of disorders. There was also no improvement in the five people who showed severe symptoms both before and after the therapy.

### 3.3. Analysis of the Influence of Selected Socio-Economic Aspects Concerning the Symptoms of Depression and Anxiety Disorders

The study group was also characterized in terms of the influence of the patient’s financial situation on the severity of D/A symptoms. The analysis shows that a worse financial situation influenced the degree of D/A symptoms (χ^2^ = 11,198, *p* = 0.024). On the other hand, the state of health (χ^2^ = 6.64, *p* = 0.363) and physical fitness (χ^2^ = 10.28, *p* = 0.113) had no effect on the clinical picture of the degree of the presented symptoms for hemiparesis caused by ischemic stroke. Subsequently, the influence of the financial situation on the results of treatment was analyzed. Statistical analysis confirmed a significant influence of the financial situation on the change in the intensity of symptoms of D/A disorders (χ^2^ = 11.83, *p* = 0.018). Similarly, the state of health (χ^2^ = 20.57, *p* = 0.022) and physical fitness (χ^2^ = 12.95, *p* = 0.044) changed the severity of symptoms of anxiety and depressive disorders.

## 4. Discussion

Stroke is considered a very serious medical problem and one of the most dangerous vascular diseases of the brain. The average incidence of stroke in Poland remains at the European level [25]. However, the information on the mortality and disability rates among this group of patients, which turns out to be one of the highest in Europe, is very disturbing [29,30]. The risk of death increases with the severity of the symptoms of depression. Such projections highlight the high risk of neuropsychiatric problems in stroke patients. Large cognitive and motor deficits resulting from damage to the brain structures and related disability, and deterioration of social and living situation and dependence on others leads to frustration, non-acceptance of self-image and the new situation. Such a negative attitude towards oneself and the treatment process delays or even makes it impossible to undertake effective rehabilitation therapy [2,31].

The research carried out in this study was performed in a 40-person study group, subjected to physiotherapeutic interventions. The results of the research indicate that 2-week physiotherapeutic rehabilitation may improve the mood and reduce the symptoms of depression in patients after a stroke. However, it cannot be unequivocally stated that it was only an effect of physiotherapeutic improvement, in particular kinesiotherapy. Additionally, in the study group, material and health conditions were characterized. The examined financial situation of the patient influenced the occurrence and severity of the clinical picture of emerging depressive and anxiety disorders. On the other hand, subjective assessment of health and physical fitness did not have such a significant impact. In turn, the change in the severity of depression was influenced by all the above-mentioned aspects, i.e., the financial situation, subjective assessment of health and physical fitness.

According to the researchers, physical activity may be as effective in treating depression in the elderly as other methods, including pharmacological treatment. Systematic physical exercise at all ages, including older ones, affects both physical and mental health, improving the quality of life [19,20,32]. On the one hand, there is a noticeable increase in vigor and vitality, and on the other, there is an effect on well-being, improving mood, while reducing the level of anxiety and depression [23,32].

In our study, after two weeks of therapy, an improvement in mood was observed in 16 (40%) patients, no changes in symptoms of anxiety and/or depression in 22 (55%) patients. Improving well-being in such a short time proves that implementing post-stroke physiotherapy is a good step that can help a stroke patient become involved in the treatment process. We also observed that two people (5%) had severe symptoms. The deterioration of mood may be a urinary tract infection in one of the patients, the persistent increase in blood pressure due to atherosclerosis seen in most patients, or the treatment of diabetes associated with intradermal insulin administration instead of oral preparations. Patients often react poorly to changes in the way insulin is administered, believing that their condition is getting worse.

With age, the level of perceived anxiety decreases, but in 2% of people over 65 years of age, symptoms of generalized anxiety appear, and over 10% of respondents report the appearance of an episode of strong anxiety in the form of a phobia. Among the respondents, anxiety is most often caused by symptoms related to material resources, serious illness and related disability, and dependence on the environment.

In our patients, after two weeks, we achieved clinical improvement and reduction in A/D symptoms. After 6 months of treatment, they were re-measured and symptoms of anxiety and depression were worse in 10% of patients than at discharge, 50% of patients had the same result and 40% improved (data not shown).

Depression is the most common mental disorder among the elderly [33]. According to Szczepańska et al. [34], systematic, general physical exercise, conducted for 3 months, every other day, for 60 min, may improve mood and reduce symptoms of depression among people over 65, in whom deficits appeared to a small extent cognitive. Thanks to the improvement in physical fitness and the reduction in pain, the anxiety associated with loss of fitness and dependence on others is reduced.

According to Guszkowska [35] the best effects are noticeable in patients participating in physical activity programs lasting several weeks. It is then observed a reduction in the level of depression and anxiety and a significant improvement in general well-being, even before a noticeable improvement in the physiological parameters of the body. As you can see, the decisive factor here is the long duration of regular physical activity.

According to available studies, it is not possible to unequivocally determine the relationship between the financial situation and the degree of symptoms, and the progress of treatment of post-stroke depression. In our research, a positive correlation was observed between these aspects. In turn, according to Martowicz and Wójcik [36] as many as 70% of stroke patients described their financial situation as good, and only 6% indicated bad. In the same study, 75% of the study group indicated physical disability as the main reason for the deterioration of the quality of life. This shows that physical fitness and the associated functional independence play a huge role. Based on the research carried out in this study, it can be concluded that health state and physical fitness do not affect the severity of the present clinical picture for hemiparesis caused by ischemic stroke, while patients who assess them better, show greater improvement during treatment. However, it is noted that the deterioration of socio-economic conditions, health, and physical fitness is a significant risk factor for depression [12,13,37].

Another issue is the rehabilitation time of patients. The longer the patient improvement period, the more reliable the research results are. In this study, a period of 2 weeks was considered, which is a relatively short time. In practice, rehabilitation programs are carried out on average for 10–12 weeks. Additionally, the group we studied was heterogeneous in terms of the nature and intensity of physical exertion and the type of exercises performed. This was mainly due to the diversity of the patients’ condition, health problems, post-stroke complications, and comorbidities [38,39].

Apart from kinesiotherapy, patients also underwent physical therapy, massage, and occupational therapy. The influence of physical factors could also, to some extent, contribute to the improvement of mood and reduction in the symptoms of anxiety and depression. Occupational therapy creates favorable conditions for patients to establish and maintain interpersonal contacts. These types of activities have a positive effect on self-esteem and self-acceptance, reduces fears and helps to adapt to new living conditions modified by the disease. They teach new ways of coping with everyday activities, at the same time showing how others function with the same disease entity [40].

Our research focused solely on assessing the impact of physiotherapeutic rehabilitation in the form of kinesiotherapy. The diagnosis of depression, especially after a stroke, is very difficult. A one-tool assessment is insufficient. Patients have repeatedly reported that the deterioration of well-being may be associated with a stay in the hospital, in a new environment, away from their relatives. This could indicate temporarily depressed well-being, not depressive or anxiety disorder.

### Limitation

Due to the widespread prevalence of depression in patients after stroke, as well as its difficult diagnosis, due to the uneven clinical picture, the research should be extended and carried out on a larger group of people. An important aspect would be the introduction of more diverse and reliable diagnostic tools. The HADS scale used in the study is insufficient as the only assessment tool for such a serious disease such as depression, especially in patients after stroke. The assessment should also include a detailed interview and medical examination. Patients with multiple cognitive deficits often reported incomprehensibility of questions, problematic answers to be given, and embarrassment related to it. As a result, the obtained results were often borderline values, and qualifying the patient to the appropriate degree of disorders was difficult and not very accurate. Moreover, it would be very useful to develop a uniform exercise program for patients and to divide patients according to the degree of presented symptoms of disorders.

The topic of diagnosis and treatment of depression is problematic and unexplained. The numerous difficulties and doubts that arose during the research are the best proof of this. It seems that there is a need for further research in this area. It would also be useful to determine which tools for the assessment of depressive disorders turn out to be the most reliable. Better diagnostics would enable research to be conducted into effective alternative treatments for this complex condition. In turn, proper prevention and treatment of depression would allow for effective treatment to rehabilitate patients after stroke. This would ultimately improve the quality of life of patients, reduce the degree of disability, and even lower the mortality rate, at least to the European average.

The main limitation of this study is the lack of a control group. We are aware of this. However, we believe all patients have the right to participate in kinesiotherapy. In our opinion, such dysfunctions as are observed after ischemic stroke, it is unethical to create a control group only for experimental purposes and deprive patients of such essential treatment as kinesiotherapy. Our study was performed to help patients in the future and had a pilot nature. We wanted to better understand their needs and expectations in order to alleviate their problems, as well as improve the therapy process and understand why therapy may be ineffective in many patients. Therefore, a control group was not used in this study.

## 5. Conclusions

Physical therapy has an impact on the course of depression in patients after stroke and might be an effective method supporting its treatment. The financial situation correlates with the severity of depressive and/or anxiety disorders and has the greatest impact on the improvement of the severity of depression. The state of health and physical fitness do not affect the severity of the present clinical picture, while patients who assess them as better have a greater improvement during treatment.

## Figures and Tables

**Table 1:** Characteristics of patients and economic and health status.

Stroke Localization		Time from Stroke to Depression (Months)			Economic Status			Health Status					Family Support		Fears about the Future				
Right Side	Left Side	Early		Late	Good	Acceptable	Bad	Very Good	Good	Accepteble	Average	Bad	Yes	No	Lack of Support	Lack of Independence	Loss of Physical Fitness	Next Stroke	Not Worried about Future
		3	6	12+															
*n* = 11	*n* = 29	*n* = 30	*n* = 2	*n* = 8	*n* = 10	*n* = 17	*n* = 13	*n* = 0	*n* = 6	*n* = 9	*n* = 20	*n* = 5	*n* = 33	*n* = 7	*n* = 1	*n* = 13	*n* = 15	*n* = 5	*n* = 6
27.5%	72.5%	80%		20%	25%	42.5%	32.5%	0%	15%	22.5%	50%	12.5%	82.5%	17.5%	2.5%	32.5%	37.5%	12.5%	15%

**Table 2:** Socio-economic status of patients.

Education			Living				Place of Living					Salary (National Average)			Living Alone
**Higher**	**Secondary**	**Primary**	**Family**	**Alone**	**City**	**Village**	**Private House**	**Block of flats**				**Above**	**Average**	**Below**	2—in a private house 8—in a block of flats: 1—on the ground floor 1—on 3rd floor
								**Ground Floor**	**1st Floor**	**2nd** **Floor**	**3rd and Higher**				
15	14	11	30	10	28	12	14	1	1	7	17	14	21	5	
37.5%	35%	27.5%	75%	25%	70%	30%	35%	10%	10%	17.5%	42.5%	35%	52.5%	12.5%	

**Table 3:** Assessment of the degree of disorders according to the HADS scale in patients before the introduction of physiotherapeutic rehabilitation.

Disorders	Score	Severity of Disorders	Number of Patients
Anxiety/Depression (A/D)	7–10	Mild	3
	11–14	Moderate	19
	15–21	Severe	5
Total			27
Anxiety (A)	7–10	Mild	5
	11–14	Moderate	3
	15–21	Severe	0
Total			8
Depression (D)	7–10	Mild	4
	11–14	Moderate	1
	15–21	Severe	0
Total			5

**Table 4:** Descriptive statistics of HADS before and after the treatments.

Disorder	Descriptive Statistics						
	N	Average	Median	Min	Max	25%	75%
						Quartile	Quartile
Anxiety before	40	982,500	1,000,000	2,000,000	1,600,000	800,000	1,150,000
Anxiety after	40	867,500	800,000	2,000,000	1,600,000	600,000	1,100,000
Depression before	40	1,000,000	1,000,000	3,000,000	1,700,000	800,000	1,300,000
Depression after	40	937,500	900,000	3,000,000	1,600,000	700,000	1,200,000

## Data Availability

The datasets generated for this study are available on request to the corresponding author.
